# Analyses of RNA-Seq and sRNA-Seq data reveal a complex network of anti-viral defense in TCV-infected *Arabidopsis thaliana*

**DOI:** 10.1038/srep36007

**Published:** 2016-10-26

**Authors:** Chao Wu, Xinyue Li, Song Guo, Sek-Man Wong

**Affiliations:** 1 NUS Graduate School for Integrative Sciences and Engineering, National University of Singapore, Singapore; 2 Vishuo Biomedical Pte Ltd, Science Park II, Singapore; 3 Department of Biological Sciences, National University of Singapore, Singapore; 4 Temasek Life Sciences Laboratory, Singapore; 5 National University of Singapore Suzhou Research Institute, Suzhou Industrial Park, Jiangsu, China

## Abstract

In order to identify specific plant anti-viral genes related to the miRNA regulatory pathway, RNA-Seq and sRNA-Seq were performed using *Arabidopsis* WT and *dcl1-9* mutant line. A total of 5,204 DEGs were identified in TCV-infected WT plants. In contrast, only 595 DEGs were obtained in the infected *dcl1-9* mutant plants. GO enrichment analysis of the shared DEGs and *dcl1-9* unique DEGs showed that a wide range of biological processes were affected in the infected WT plants. In addition, miRNAs displayed different patterns between mock and infected WT plants. This is the first global view of *dcl1-9* transcriptome which provides TCV responsive miRNAs data. In conclusion, our results indicated the significance of DCL1 and suggested that PPR genes may play an important role in plant anti-viral defense.

Plants develop a complex and effective defense system to resist pathogen infection during evolution. The conserved pathogen-associated molecular pattern (PAMPs) is participated in the first layer of the defense system, where the PAMP-triggered immunity (PTI) is initiated to prevent spreading of pathogens. Then an effector-triggered susceptibility (ETS) is started to respond to the effector proteins delivered by invading pathogens. Accordingly, plants subsequently evolved resistance (R) proteins or R genes in response to the effector proteins. This immunity is called ‘effector-triggered immunity’ (ETI), more rapid and robust that leads to disease resistance^1^.

Plant viruses are pathogens which infect plant cells and cause systemic symptoms. To explore the underlined mechanism of plant anti-viral system, a number of studies have been carried out in different plant species after virus infection to identify the virus-responsive transcriptomes^2^,^3^,^4^,^5^,^6^. Some of the gene expressions are common, while others are virus-specific. Belonging to the *Carmovirus* family, Turnip crinkle virus (TCV) is a positive-strand RNA virus that can infect *Arabidopsis*. Most *Arabidopsis* ecotypes are highly susceptible to TCV, except for the TCV resistant line Di-17 derived from ecotype Dijon. Inoculation of TCV in Di-17 results in necrotic lesion formation and a hypersensitive response on the inoculated leaves, while no disease symptoms were observed on the un-inoculated portions of most plants^7^,^8^. Five open reading frames are identified in the TCV genome^9^. The virus replication protein p28 and its read through product p88 are RNA-dependent RNA polymerases that are responsible for virus replication^10^. The movement proteins p8 and p9 help virus move from cell-to-cell^11^. The coat protein p38 enables the capsidation of virions and help to facilitate systemic virus movement^12^,^13^. It also acts as a gene silencing suppressor in plant defense^14^. Previous study had analyzed the transcriptome of TCV-infected Arabidopsis. Many of the stress related genes have changed significantly after TCV infection^15^.

Besides the virus-triggered genes, small RNAs also play critical roles in plant defense by triggering either transcriptional and/or post-transcriptional gene silencing. In addition to the siRNAs that are generated by virus infection, endogenous miRNAs are also important. With sizes of ~18–25 nucleotides, miRNAs are thought to function in diverse processes, including cellular differentiation and apoptosis, binding to targets and controlling the expressions of target genes^16^.

The miRNAs are generated from their own primary transcription units (pri-miRNAs), with their lengths range from hundreds to thousands nucleotides. The pri-miRNAs contain an intronic or exonic stem-loop secondary structure, where the mature miRNAs locate in one of the stems. Briefly, processing of pri-miRNAs to mature miRNAs involves three steps: first cleavage, second cleavage and strand collection^17^. In plants, both of the two cleavages occur in nucleus and guided by Dicer-like protein 1 (DCL1). DCL1 first cleaves the cap and the lower stem of the pri-miRNAs to produce a pre-miRNA and then cleaves the pre-miRNAs to release the miRNA/miRNA* duplex which is then exported to cytoplasm. One strand of the duplex, the mature miRNA, is incorporated into AGO1 to target the genes of interest^18^. Disruption of the DCL1 leads to increased cell division in floral meristem^19^, accumulation of miRNA precursors and failure of miRNA production^20^. Consequently, a series of developmental defects appeared in weak or null dcl1 mutants^21^. The weak dcl1 alleles, like *dcl1-7* (*sin1-1: short integument1-1*) and *dcl1-9* (*caf-1: carpel factory-1*) display phenotypes such as small leaves, late flowering and female sterility. Whereas null mutant, like *dcl1-5* (*sus1-5: suspensor1-5*), is embryonic lethal. The loss of mature miRNAs production associated with developmental defects imply that most if not all miRNAs are indispensable determinants for plant development. Although the *dcl1-7* and *dcl1-9* are both weak mutants, their miRNA expression profiles showed differences. In the 12 conserved miRNAs tested, almost all of the miRNAs abundances are reduced significantly. The levels of some miRNAs (miR156, miR159, miR162 and miR172) are decreased more significantly in *dcl1-9* than in *dcl1-7*^22^. Therefore, *dcl1-9* was chosen for our study. DCL1 plays an important role in conferring infections caused by plant pathogens in general^23^,^24^,^25^,^26^,^27^. It also acts as a negative regulator of DCL3 and DCL4, resulting in repression of antiviral RNA silencing^28^,^29^.

In addition to the regulation of plant development, miRNA could have a direct function in viral defense. In plants, RNA silencing is a critical innate immune approach to fight against viruses. After virus infection, small interfering RNAs (siRNAs) are generated by RNA interference and involved directly in viral resistance. Most of the plant miRNAs target transcription factors^30^. Bioinformatics analysis also shows that miRNAs can potentially target virus genome directly^31^. However, the specific roles of plant miRNAs in TCV resistance are unknown.

In this study, TCV-infected WT *Arabidopsis thaliana* and *dcl1-9* mutant plants were selected for the high throughput transcriptome and small RNA (sRNA) analysis. Thousands of host genes and 17 miRNA families were triggered by TCV infection. In addition, 32 novel miRNAs were predicted. Using *dcl1-9* mutant, we showed that significantly less host defense genes were triggered when DCL1 functions were blocked.

## Results

### TCV replication level between WT and *dcl1-9* plants

**Figure 1:**
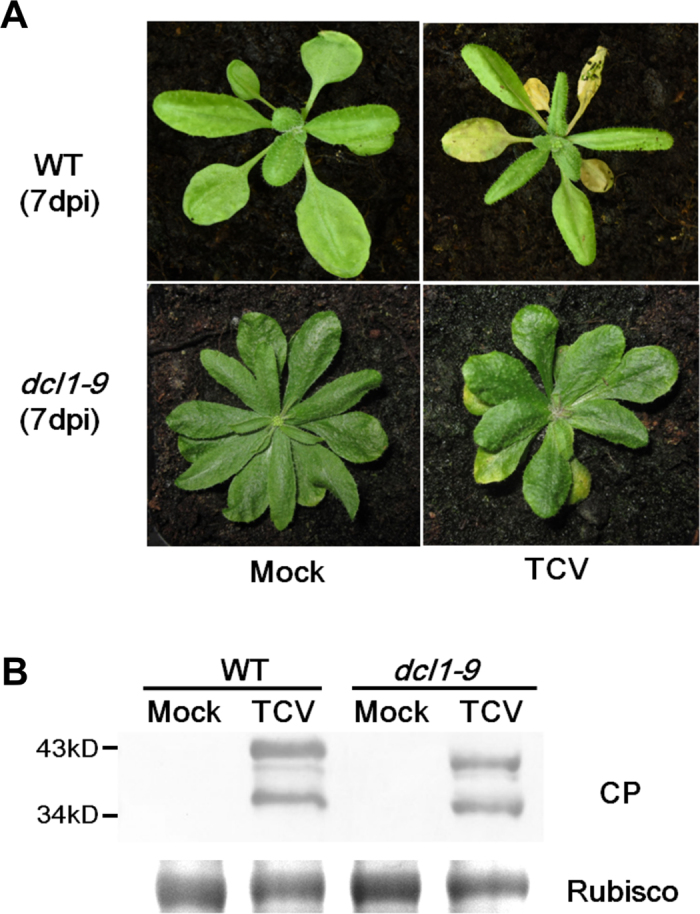
TCV replication level between WT and *dcl1-9* mutant. (**A**) Both WT and *dcl1-9* mutant plants displayed TCV symptoms at 7 dpi. (**B**) Virus replication levels were estimated by Western blot. The intensities of protein bands were quantified by ImageJ software. Rubisco was selected as a loading control.

Both WT and *dcl1-9* plants showed chlorotic symptoms at 7dpi of TCV inoculation (Fig. 1A). The TCV CP expression levels in WT and mutant were not significantly different (Fig. 1B), as determined by the student’s *t* test (p value = 0.3056, at 95% confidence interval).

### Data processing of transcriptomes

Using the Illumina HiSeq 2000 platform, a total of more than 1.8 billion clean reads were generated from all four cDNA libraries. Of these, 86.6% and 85.73% for WT plants, 78.33% and 80.17% for *dcl1-9* mutant plants were mapped to the *Arabidopsis* reference genome. In the infected mutant plants, the virus mapping rate is lower (1.73%) when compared to that in infected WT plants (7.19%). A summary of data quality, filtration and alignment statistics obtained for each sample is presented (Table 1). To validate the RNA-Seq data, the relative expression levels of selected genes from the up-regulated, down-regulated and non-significant changed gene category (the entire gene list can be found as Supplementary Tables S1 and S2) were tested by real time PCR (see Supplementary Fig. S1).

**Figure 2:**
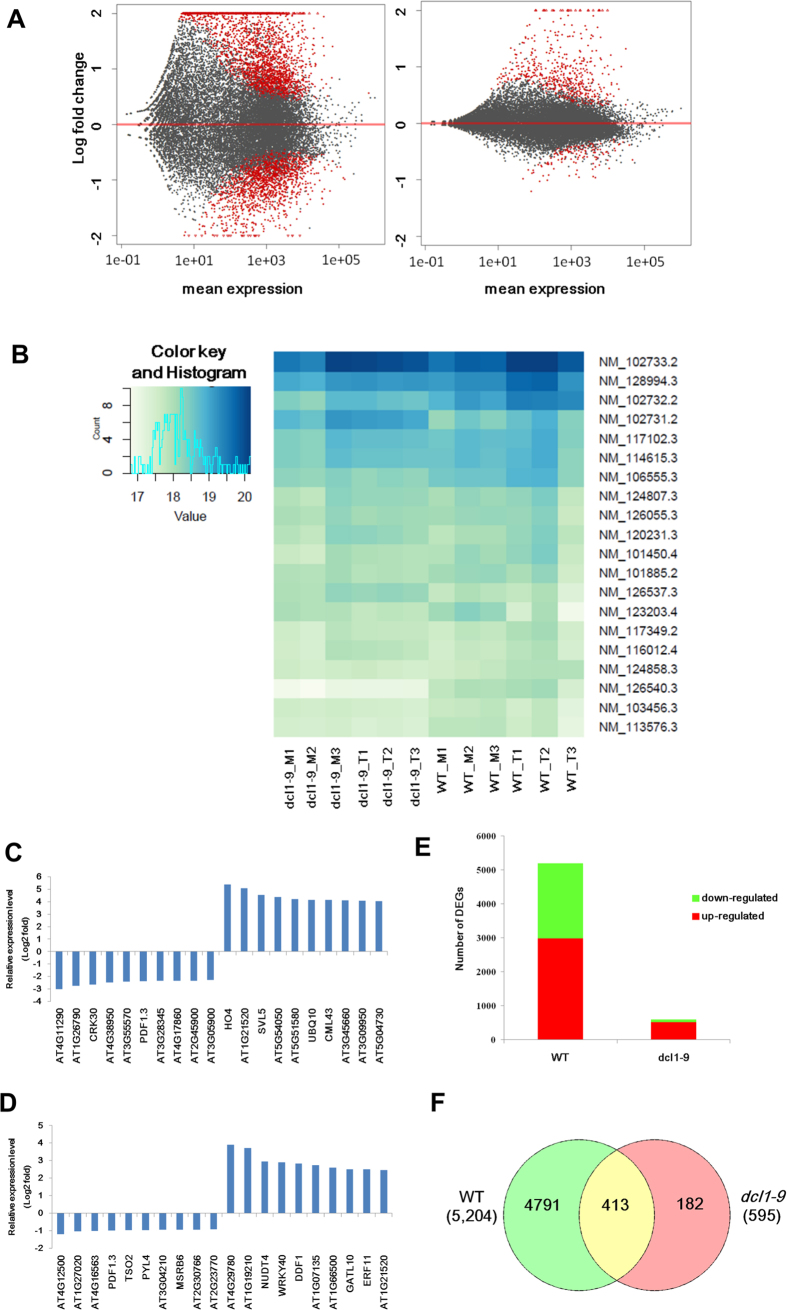
Distribution of differentially expressed genes (DEGs). (**A**) MA-plots, showing the comparisons of global gene expression profiles. Left, WT; right, *dcl1-9* mutant. Each gene is represented as a dot. The red dots represent DEGs (P*adj* < 0.05, >1.5 fold); y-axis represents log2 fold change; x-axis represents average counts (mean expression). (**B**) Heatmap of the top 20 DEGs. (**C,D**) Relative expression levels of the top 20 DEGs of WT (**C**) and *dcl1-9* (**D**). (**E**) Numbers of up-regulated and down-regulated DEGs in WT and *dcl1-9*. (**F**) Venn diagram of WT and *dcl1-9* shared DEGs (yellow) and *dcl1-9* unique DEGs (pink).

To provide an overview of the transcriptomes, MA plots and heatmaps (Fig. 2A,B) were generated. The top ten genes significantly changed were shown (Fig. 2C,D). Statistic numbers of the up-regulated and down-regulated genes in each comparison were displayed in a bar chart (Fig. 2E). Compared to the total number of genes changes from the WT plants (5,204), fewer genes (595) were showed to be abrogated in the mutant plants after TCV infection.

### Identification of differentially expressed genes (DEGs) and gene ontology (GO) enrichment analysis

**Figure 3:**
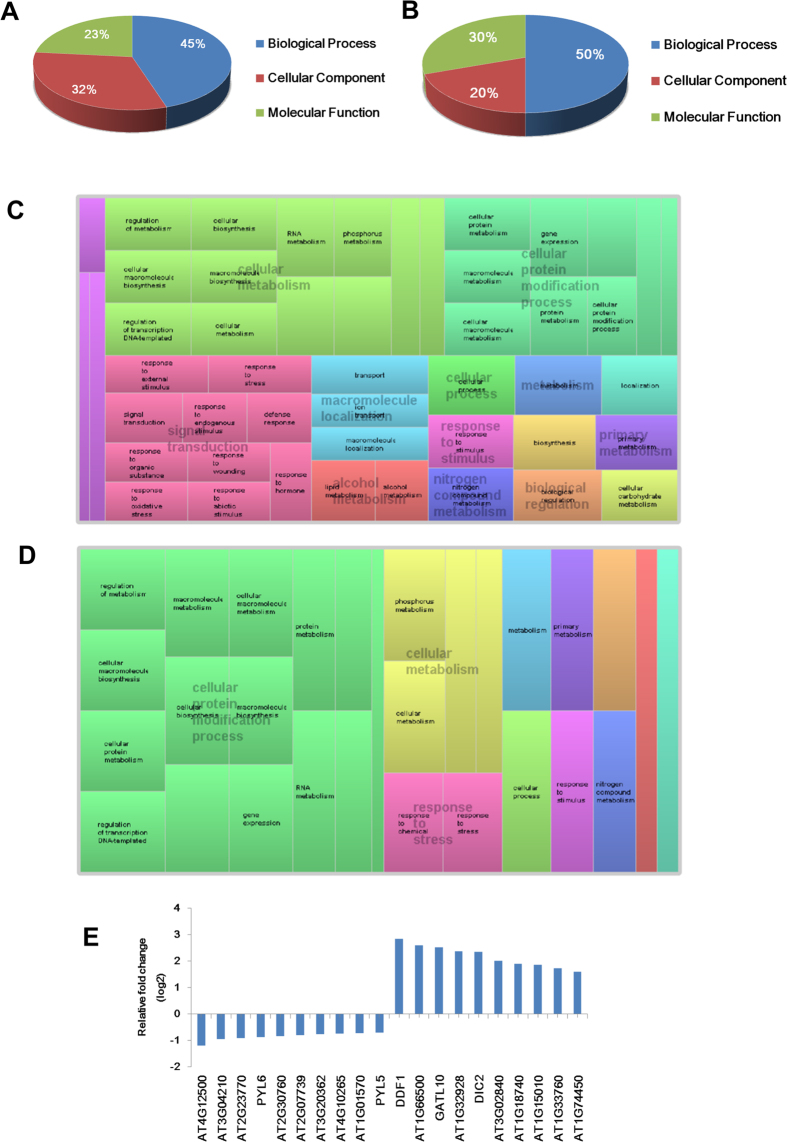
GO analysis of WT and *dcl1-9* shared DEGs and *dcl1-9* unique DEGs. Fraction distributions of WT and *dcl1-9* shared DEGs related GO terms (**A**) and *dcl1-9* unique DEGs related GO terms (**B**) based on three main functions. Treemap visualization of GO biological process terms for the shared (**C**) and unique DEGs (**D**). (**E**) Relative expression levels of the top 10 up-regulated and top 10 down-regulated *dcl1-9* unique DEGs.

In order to identify the TCV infection responsive genes, data collected from mock and infected WT plants were compared (WT_T vs WT_M). Genes with fold-change greater than 1.5 fold and P_*adjust*_ value (P_*adj*_) less than 0.05 were considered as differentially expressed genes (DEGs)^32^. A total of 5,204 DEGs (2,977 up-regulated and 2,227 down-regulated) were found in the infected WT plants. Comparing mock and infected *dcl1-9* mutants, the number of DEGs in the infected mutant was 595 (518 up-regulated and 77 down-regulated), which is much lower compared to WT plants. It implies that significantly fewer genes were affected when DCL1 function was abrogated after TCV infection. Among these DEGs, a majority of them (413 out of 595) were overlapped in both WT and mutant, which were considered to be common TCV responsive genes, whereas the rest (182) were uniquely found in *dcl1-9* mutant (Fig. 2F). The entire lists of genes can be found as Supplementary Tables S3 and S4. The top 10 up-regulated and top 10 down-regulated *dcl1-9* unique DEGs and their relative expression levels were shown in Fig. 3E.

To further explore the distribution of DEGs, gene ontology (GO) enrichment analyses were performed with these DEG sets. For the shared TCV responsive genes (overlap in WT and mutant), a total of 140 GO terms were classified into biological process (45%), cellular components (23%) and molecular function (32%) (Fig. 3A). To display the correlations of the interesting biological process GO terms, treemaps for shared or *dcl1-9* unique GO terms were shown (Fig. 3C,D). A wide range of biological processes were affected. The most affected processes were cellular metabolism, cellular protein modification and signal transduction (Fig. 3C). For the 92 unique GO terms in *dcl1-9* mutant, the percentages of the three functional classes were 50%, 30% and 20%, respectively (Fig. 3B). The biological processes which were most affected in the *dcl1-9* mutant was similar to that in WT, except for the signal transduction that was replaced by response to stress (Fig. 3D). The entire lists of the shared and unique GO terms can be found as Supplementary Table S5.

### Data processing of small RNAs

**Figure 4:**
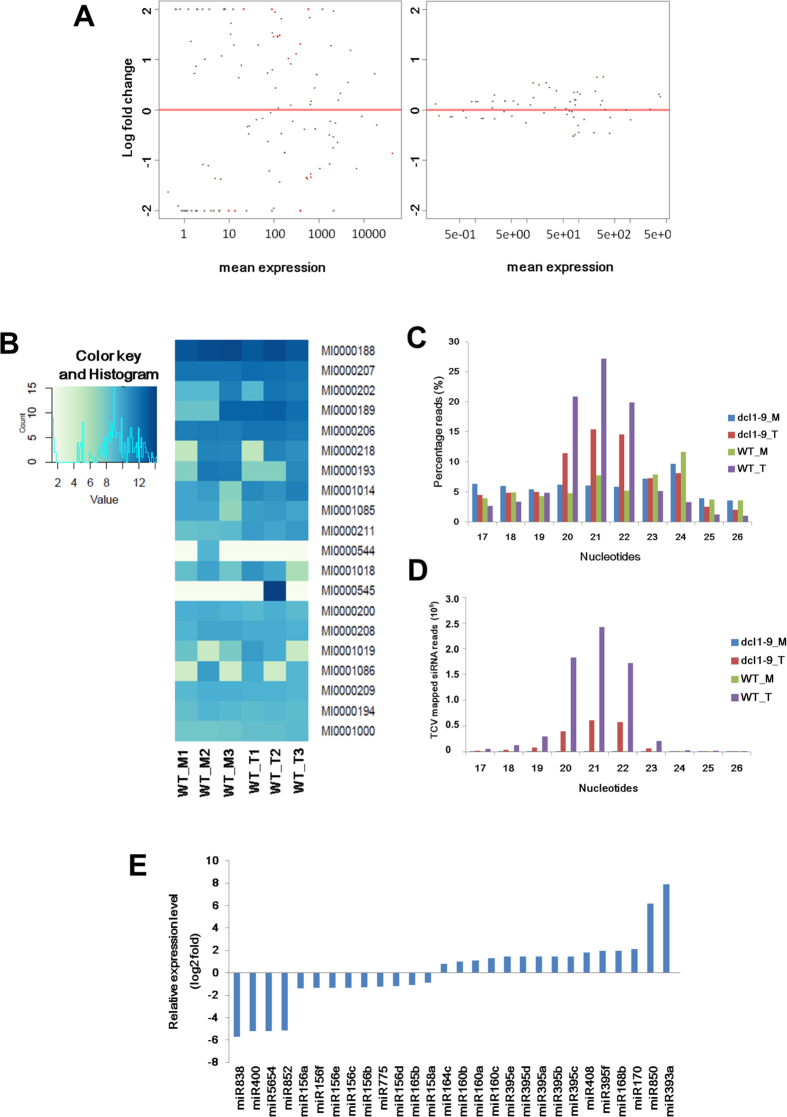
Distribution of differentially expressed miRNAs showed in MA plot (**A**) and heatmap (**B**). MA-plots, showing the comparisons of global miRNAs expression profiles. Each miRNA is represented as a dot. The red dots represent differentially expressed miRNA (P*adj*< 0.05, >1.5 fold); y-axis represents log2 fold change; x-axis represents average counts (mean expression). (**C**) Length distribution of sRNAs. (**D**) Length distribution of vsiRNAs. (**E**) Relative expression levels of the differentially expressed miRNAs.

To identify the sRNAs that response to TCV infection, data collected from TCV-infected WT (WT_T) plants, mock WT plants (WT_M), TCV infected *dcl1-9* mutant plants (*dcl1-9*_T) and mock *dcl1-9* mutant plants (*dcl1-9_*M) were used to construct small RNA libraries. After removal of the adaptor sequences and low-quality reads, and filtration of some contaminant reads, the clean reads of each library were calculated accordingly (Table 1) and subsequently mapped to *A. thaliana* reference genome and TCV genome via Bowtie2. The average mapping rates to the TCV genome were tabulated to confirm the successful infection in the infected plants. Similar to transcriptome, the virus mapping rate in infected mutant plants was lower (52.63%) than that in infected WT plants (87.46%). The length distribution of small RNA sequences ranged from 17 nt to 26 nt (Fig. 4C). The small RNA patterns in WT plants were similar to that in mutant plants. After virus infection, in both of WT and *dcl1-9* mutant plants, the abundance of sRNAs with lengths ranging from 19 nt to 22 nt were increased significantly. Virus-generated siRNAs might attribute to higher percentages. For sRNA with the size of longer than 22 nt, the abundances in mock were higher than virus-infected plants. In virus-infected plants, the most abundant size is 21 nt (27.17% in WT and 15.4% in virus-infected plants), followed by 20 nt. In mock plants, the most abundant sizes were 24 nt and 23 nt, respectively. In comparison, the percentages of sRNAs in mutant plants were smaller than that of the WT. To further investigate the vsiRNAs expression pattern, we differentiated viral small interfering RNAs (vsiRNAs) reads from total sRNA reads and analyzed the size distribution in the mutant and WT plants (Fig. 4D). The vsiRNAs in mutant and WT showed different length distribution patterns. In the mutant, the most abundant sizes were 21 nt, 22 nt and 20 nt, respectively. In WT samples, the sizes were 21 nt, 20 nt and 22 nt, respectively.

### TCV infection responsive miRNAs

Since the vsiRNAs in *dcl1* mutants was investigated previously^28^, we focused on the miRNAs that are involved in the anti-TCV response. To explore the miRNAs that differentially expressed in response to TCV infection, normalized read counts of miRNAs with p < 0.05 from WT_T with WT_M library were compared. The miRNAs with *P*_*adj*_ value < 0.05 were identified as differentially expressed in response to TCV infection. MA plots and heatmap generated with the sRNA-Seq data provided an overview of the differential sRNA expression patterns (Fig. 4A,B). A total of 30 miRNAs species, which clustered into 17 families, were identified as respond to TCV infection (Table 2, Fig. 4E).

The abundance of miR160, miR168, miR170, miR393, miR395, miR408 and miR850 were specifically increased. On the contrary, miR156, miR158, miR164, miR165, miR400, miR5654, miR775, miR829, miR838 and miR852 were down-regulated by TCV infection. To investigate their potential functions after virus infection, their predicted and verified target genes were analyzed. Most of the targets identified have been reported to be involved in development and stress responses, which are closely related to plant immune system.

### miRNAs responsive to DCL1 deficiency and prediction of novel miRNAs

As a DCL1 deficient mutant, the cleavage efficiency of DCL1 in *dcl1-9* was poor. Attributed to this defect, almost all of the miRNAs in the *dcl1-9* mutant were down-regulated. To compare the expression patterns of miRNAs and their targets, sRNA-Seq and RNA-Seq data were combined for analysis. As expected, most targets displayed opposite expression trends compared with the corresponding miRNAs. For example, miR395 was induced after virus infection in WT, the expression of its targets: adenosyl phosphatosulfate kinase (APS) 1, 2 and 4 and sulfate transporter 3;5 (SULTR3;5) were subsequently reduced. Relative expression levels of selected miRNAs and their targets were verified by real time PCR (Fig. S2).

From the four sRNAs libraries, a total of 94 mature miRNA sequences were predicted. To narrow down the list, only those with total read counts more than 10 were selected and tabulated (Table 3).

## Discussion

In *dcl1-9* mutant, the CP expression level was not significantly different from that of the WT. This result is in agreement with previous report, in which the TCV accumulation level does not change in *dcl1-7* mutant^28^.

The accelerated development of high throughput sequencing approach allows identification of transcriptome and sRNAs in *Arabidopsis* and other plant systems. In addition to comparison of gene expression under different treatments, the RNA-Seq and sRNA-Seq enable novel genes expressed at low levels to be profiled, which could not be achieved by traditional sequencing methods. Our sequencing results obtained from infected and non-infected WT and *dcl1-9* mutant provided a global view of mRNAs and sRNAs expression pattern in *Arabidopsis* leaves. The lower virus mapping rate in the mutant showed that there was apparent lesser amount of CP transcripts. This observation supported the conclusion that DCL1 plays a negative role in anti-TCV response^28^.

DCL1 is known to play a key role in PTI^33^. But its role in ETI is unknown. In this report, we want to investigate the role of DCL1 in ETI by analyzing the ETI related gene expression patterns. For ETI, a number of common gene sets were identified after virus infection in diverse plant species. Their functions are mainly involved in defense response, cellular stress response and developmental process. The TCV responsive genes in WT were identified in previous report^15^, reporting that most of the DEGs are stress related or immune response related. The defense response genes are depicted by the induction of pathogenesis related (PR) genes and other plant disease defense related genes, while stress response genes by the induction of heat shock proteins (HSP). In *Arabidopsis*, the defense responsive genes include the PR gene family (PR-1, PR-2, PR-3, PR-4 and PR-5), the glutathione S-transferase (GST) gene family and the WRKY gene family. In our results, PR-1 and PR-5 were increased after TCV infection in WT plants. Similarly, Cucumber mosaic virus (CMV) and Oilseed rape mosaic virus (ORMV) infection in *Arabidopsis* also induce PR-1^34^. The PR-1 induction was accompanied with the up-regulation of NPR1, NPR2 and NPR3. In *dcl1-9* mutant plants, only NPR3 expression was changed significantly, while none of the PR genes was induced, suggesting that signaling pathway was abrogated or initiation of the pathway is delayed in *dcl1-9* mutant. Noticeably, plant defensin 1.3 (PDF1.3), which is predicted to encode a PR protein, was listed among the top 10 down-regulated genes in both WT and mutant plants. The coincidence of PDF1.3 expression in both samples suggested that it may act as a key PR gene after TCV infection. The jasmonic acid (JA) signaling pathway is an important plant defense fine tuner which is mediated through the NPR1 expression mechanism^35^. The downstream JA responsive defense genes include several transcription factors such as ethylene transcription factor (ERF), basic helix-loop-helix (bHLH) and WRKY transcription factors. In our RNA-Seq results, ERF2, ERF4, ERF5 and ERF6 are induced in TCV infected samples. Besides, ORA47, a key regulator of JA biosynthesis in *Arabidopsis*, was also increased after TCV infection in both WT and *dcl1-9* mutant plants. The expressions of ERF genes and ORA47 shared the similar trends, whereas the fold changes in *dcl1-9* mutant plants were higher than that of WT, indicating that *dcl1-9* mutant possessed a stronger JA-mediated defense against TCV infection. In addition to the ERF genes and ORA47, the WRKY genes, a type of genes that are induced after virus infection, displayed a different expression pattern after TCV infection. A total of 24 WRKY genes were identified to have their abundance changed significantly in WT plants. While in *dcl1-9* mutant, eight WRKY genes were changed significantly. These results implied that there may be alternative pathway regulating the induction of defensive related WRKY genes, when the function of DCL1 protein was abolished. From our RNA-Seq results, a common set of HSP genes were induced after TCV infection in WT plants: HSP17.4, HSP17.6, HSP23.6, HSP70b, HSP83, HSP89.1 and HSP101, respectively. However, only HSP89.1 (synonyms: HSP90.6) was induced in *dcl1-9* mutant after TCV infection. Although the expression of *Arabidopsis* HSP89.1 was barely induced by heat shock^36^, the presence of this gene in both WT and mutant datasets suggests that it is virus responsive. In addition to the common virus-responsive genes, we also found the TCV-specific HRT-mediated resistance gene called compromised for recognition of TCV (CRT1), which is required for ETI^37^,^38^, displayed different expression patterns in WT and *dcl1-9* mutant plants. In TCV-infected WT plants, the CRT1 expression level was significantly reduced; while in the infected *dcl1-9* mutant, CRT1 was not detected. These results suggested that DCL1 may be involved in regulating ETI. However, such deduction is solely based on bioinformatics prediction. Further experiments are needed to verify the prediction.

From our vsiRNAs size distribution results, 21 nt is the major size, which is different from previous result^39^, but in consistent with other sRNA-seq reports that performed in other types of viruses^40^,^41^. This indicates the major size of vsiRNAs may vary among different viruses. Although the TCV transcripts present in the mutant was lower, the amount of vsiRNAs generated in *dcl1-9* mutant was relatively high (Table 1). It indicates that the vsiRNA processing rate was more efficient than that of the WT. Since DCL1 is a negative regulator in DCL4-induced anti-viral RNA silencing pathway, when DCL1 is disrupted, it allows other DCLs to generate more vsiRNAs.

Our report provides a global view of TCV responsive miRNAs. According to our results, most of the TCV infection responsive miRNAs have been known to response to either biotic or abiotic stress in diverse plant species. Both miR393a and miR160 are auxin pathway regulators, and they were significantly up-regulated after TCV infection. As a result, their targets, auxin response factor TIR1, AFB2, AFB3, ARF10 and ARF16 were repressed. Four of the TCV responsive miRNAs are with unknown functions (miR850, miR829, miR838, miR852). Potential targets of these miRNAs were predicted by online tool psRNATarget^42^. The miR838 is located in the intron 14 of DCL1, which enables a self-regulation of DCL1^43^. In our results, miR838 showed a significant decrease after TCV infection, indicating it is TCV related and it may involve in the plant anti-viral defense pathway. It may either be due to regulatory feedback of DCL1 or other mechanism. The other three miRNAs (miR850, miR829 and miR852) that were affected by TCV infection, implies that their potential target genes may also be involved in the host anti-viral defense system.

Pentatricopeptide Repeat (PPR) proteins are a large family proteins contain tandem repeated degenerate 35 amino acid sequence motif (PPR motif)^44^,^45^. Varied in the PPR motif numbers, the PPR proteins are mainly involved in RNA editing^46^, transcript processing^47^ and translation initiation^48^. In *Arabidopsis*, there are 441 identified PPR genes^49^. In our results, three TCV responsive miRNAs (miR158, miR400 and miR5654) which target PPR genes were decreased after TCV infection. The down-regulations of these miRNAs were correlated to the increasing abundance of their target genes. These miRNAs and their regulations to the targets were showed to be associated with stress. For example, miR158 was decreased when the plants were under nitrogen starvation^50^, miR5654 and its target AtPPC3 were shown to be nitrogen-responsive when the plants were treated with nitrogen^51^. The miR400 was down-regulated, whereas its targets, PPR1 and PPR2, were up-regulated when the plants were challenged with pathogenic bacteria or fungi^52^. In addition to these miRNA targets, other PPR encoding genes were also found to be abrogated after TCV infection. One of these genes, dwarf and delayed flowering 1 (DDF1), showed highest fold change in TCV-infected *dcl1-9* mutant, suggesting there may be other mechanism to control the PPR protein expression other than miRNA regulation. Taken together, this is the first time to show that PPR genes may play an important role in plant anti-viral defense. For future study, a number of candidate miRNAs targets will be investigated after TCV infection. Our results also provided insights for further research on host-virus interactions.

## Conclusion

This is the first summary of TCV responsive transcriptomes and miRNAs in relation to virus defense pathways. There is a significant reduction of DEGs in *dcl1-9* mutant, highlighting DCL1 plays a negative role in anti-TCV response. In addition, PPR genes may play an important role in plant anti-viral defense.

## Methods

### Plant materials and virus inoculation

*Arabidopsis thaliana* ecotype Col-0 (WT) and *dcl1-9* heterozygous plants were grown in plant growth chambers at 22 °C with 18 h light and 6 h dark cycles. DCL1-9/DCL1-9 homozygous plants were distinguished from DCL1-9/dcl1-9 heterozygous plants by PCR amplification with the primers 5′-CTCCGTTCAATTTACTGATTGTAC-3′ and 5′-TTGAATGGTGCCCGTAACTTTCG-3′^53^ using genomic DNA as the templates. The pT1d1 (a full length cDNA clone of TCV)^9^ was obtained from Dr. Jack Morris of University of Nebraska, Lincoln, USA. *Nicotiana benthamiana* leaves were inoculated with *in vitro* transcripts of pT1d1 to generate TCV virions for purification accordingly^15^. A total of 5 ug of purified TCV particles suspended in phosphate buffer containing 50 mM Na_2_HPO_4_ [pH 7.0] and 1% Celite^®^ were inoculated to a 4-weeks-old plant. For the mock plants, equal volume of phosphate buffer was used for inoculation. In order to minimize experimental variations, all leaf samples were consisted of pools of six leafs collected from three inoculated plants. All experiments were repeated three times.

### Protein extraction and western blot

Systemic leaves and inoculated leaves from the inoculated plants at 7 dpi were collected and palverized with mortar and pestle using liquid nitrogen to release total proteins from cells. Cold protein extraction buffer^54^ was added into the powder proportionally. The cell debris was removed by centrifuging samples at 12,000 × *g* for 20 min at 4 °C. Protein samples were then separated on a 12% SDS PAGE gel and transferred onto a nitrocellulose membrane. The membrane was incubated with anti-TCV CP antibody, followed by goat anti-rabbit secondary antibody, and visualized after adding NBT/BCIP (Fermentas).

### RNA extraction, reverse transcription and quantitative real time PCR

Twelve leaf samples from four different groups: mock WT (WT_M), TCV infected WT (WT_T), mock mutant (*dcl1-9*_M) and TCV infected mutant (*dcl1-9*_T) were collected for RNA extraction. Each group contained three biological repeats, in which leaves from four individual plants were pooled together. Total RNA were extracted using Trizol^®^ reagent (Invitrogen). The integrity of these RNA samples were checked by running 0.8% agarose gel electrophoresis. Each RNA sample was measured by NanoDrop^®^ at OD_260 nm_/OD_280 nm_ and Agilent 2100 Bioanalyzer, respectively. Total RNA of each sample (2 μg) was reversed transcribed to cDNA with oligo (dT) primer using SuperScript^®^ III Reverse Transcriptase kit (Invitrogen). Fragments of selected genes were amplified with appropriate primers listed in Supplementary Table S6. Expression levels of the selected genes were analyzed via real-time PCR. *Arabidopsis* tubulin gene was used as an internal control. The expression level of miRNAs were tested by stem-loop RT-PCR according to the stem-loop RT-PCR protocols^55^, using miRNA mature-sequence-specific primers listed in Supplementary Table S6.

### RNA-Seq library construction, sequencing and data analysis

For RNA-Seq, mRNA was enriched from total RNA by Oligo (dT) beads and rRNA was removed by the Ribo-Zero rRNA Removal Kit (Plant Leaf) kit (Illumina). The mRNA was subsequently fragmented randomly by adding fragmentation buffer. Library construction was performed as per standard protocol of NEBNext^®^ Ultra Directional RNA Library Prep Kit for Illumina. Paired-end sequencing was performed on Illumina HiSeq 2000 with read length of 150 bp. The raw reads containing low quality and reads and adaptors were filtered to obtain clean reads. Subsequent bioinformatics analyses were performed with clean reads according to the following pipeline: clean reads were aligned to the *A. thaliana* reference genome by Tophat^56^, the mapped reads were manipulated to BAM files by SAMtools^57^, then calculated the gene expression level by HTseq^58^. Differentially expressed genes were acquired by DESeq2^59^; the unmapped BAM files were converted to Fastq files via bedtools and aligned to virus reference genome by Bowtie 2^60^.

### Small RNA libraries construction, sequencing and data analysis

After quality control tests, the small RNA libraries were constructed by Small RNA Library Prep Kit for Illumina. Single-end sequencing was performed on Illumina HiSeq 2000 with read length of 50 bp. To analyze the known miRNAs in each library, the clean sequencing data were aligned to *A. thaliana* reference genome by Bowtie2. Known miRNAs were analyzed by miRDP^61^ and differentially expressed miRNAs were acquired by DESeq2. For vsiRNAs analysis, the total sRNAs clean reads were mapped to TCV genome by Bowtie2. The completely mapped sRNAs seqences were termed as vsiRNAs that were generated from virus after infection.

## Additional Information

**Acession codes**: Raw sequence data are available through NCBI’s Sequence Read Archive (Gene Expresion Ominbus, accession number: GSE85070).

**How to cite this article**: Wu, C. *et al*. Analyses of RNA-Seq and sRNA-Seq data reveal a complex network of anti-viral defense in TCV-infected *Arabidopsis thaliana*. *Sci. Rep*. **6**, 36007; doi: 10.1038/srep36007 (2016).

**Publisher’s note**: Springer Nature remains neutral with regard to jurisdictional claims in published maps and institutional affiliations.

## Supplementary Material

Supplementary Information

Supplementary Table S1

Supplementary Table S2

Supplementary Table S3

Supplementary Table S4

Supplementary Table S5

Supplementary Table S6

## Figures and Tables

**Table 1:** Data quality, filtration and alignment summary for transcriptome and sRNA sequencing of TCV infected *dcl1-9* mutant and WT plants.

Sample	Error rate (%)	Raw reads	Clean reads	Ave overall mapping rate to virus	Ave overall mapping rate to host
RNA-seq					
* dcl1-9*_M	0.03	47155365	46712485	0.00%	78.33%
* dcl1-9*_T	0.03	47105814	47086893	1.73%	80.17%
* *WT_M	0.03	47249649	47027652	0.19%	86.60%
* *WT_T	0.03	46493706	46186167	7.19%	85.73%
sRNA-seq					
* dcl1-9*_M	0.01	23404642	22422124	0.54%	63.12%
* dcl1-9*_T	0.01	22075148	21033182	52.63%	52.27%
* *WT_M	0.01	22873226	22026042	0.19%	58.76%
* *WT_T	0.01	22645661	21997229	87.46%	22.37%

**Table 2:** Differentially expressed miRNAs in response to TCV infection.

	miRNA species	miRNA family	Targets
Abundance increased	miR160a-3p/miR160a-5p	miR160	Auxin response factors
	miR160b		
	miR160c-3p/miR160c-5p		
	miR164c-3p/miR164c-5p	miR164	NAC domain containing proteins
	miR168b-3p/miR168b-5p	miR168	AGONAUTE1
	miR170-3p/miR170-5p	miR170	GRAS domain or SCARECROW-like protein
	miR393a-3p/miR393a-5p	miR393	F-box protein; bHLH transcription factors
	miR395a	miR395	ATP sulphurylases
	miR395b		
	miR395c		
	miR395d		
	miR395d		
	miR395e		
	miR395f		
	miR850	miR850	unknown (AtSweet4*)
Abundance decreased	miR408-3p/miR408-5p	miR408	Peptide chain release factor; plantacyanin
	miR156a-3p/miR156a-5p	miR156	SBP family of transcription factors
	miR156b-3p/miR156b-5p		
	miR156c-3p/miR156c-5p		
	miR156d-3p/miR156d-5p		
	miR156e		
	miR156f-3p/miR156f-5p		
	miR158a-3p/miR158a-5p	miR158	Pentatricopeptide Repeat (PPR) protein, At3g03580
	miR165b	miR165	Class III HD-ZIP transcription factors
	miR400	miR400	Pentatricopeptide Repeat (PPR) Protein, At1g06580 & At1g62720
	miR5654-3p/miR5654-5p	miR5654	Pentatricopeptide Repeat (PPR) Protein, AtPPC3
	miR775	miR775	Galactosyltransferase family protein
	miR829-5p	miR829	unknown
	miR838	miR838	unknown (DCL1*; At2g45720*)
	miR852	miR852	unknown (TIR1*)

^*^predicted, targets not-validated.

**Table 3:** Novel miRNAs discovered in this study.

Chr	Strand	Sequence
1	−	UAGCUAAGGAUUUGCAUUCUC
1	+	UGGAAGAUGCUUUGGGAUUUAUU
1	+	GUGAAUGCUCUGUAAA
1	+	UGUGAAUGCUCUGUAAA
1	−	UUAUGUGAUACAUUGACCUCC
1	+	UUAGAGAGUUGUAGG
1	+	AGCUAAGGAUUUGCAUUCUC
1	−	AGCUAAGGAUUUGCAUUCUC
2	−	AGAGGUGACCAUUUGAACAUAAUG
2	+	UGAGAUGAAAUCUUUGAUUGG
2	+	AAAUAGACUUAGAUAG
2	+	AAUAGACUUAGAUAG
2	+	UUUUACUGCUACUUGUGUUCC
2	−	CUUAUGUGUUGCUGCUU
3	+	CUCAUGUUGCAUCCCU
3	+	AGUGACUGUUGAUUUAGU
3	−	AGUGACUGUUGAUUUAGU
3	+	UUGUGCGGUUCAAAUAGUAAC
3	−	AUAUUUUCGUUACUAUUUGAACCG
4	−	AAAAGACUUUGAAAAG
4	−	AAAGACUUUGAAAAG
4	+	ACCGCGGACGGUGUUCUUGAUUGU
4	−	UACGUUGUUGCAGUU
4	+	AUUGUGCUUUGAAUAAUAAUA
5	+	AAUUGUCAGUAUAAAUCUUUGAUC
5	−	GUGGAUUGUGAAUUC
5	+	AAUUGUCAGUAUAAAUCUU
5	+	AGUGACUGUUGAUUUAGU
5	+	UAAGUUAAGAUUUGUGAAGAA
5	−	AGAUCGAUAAACCUCUGCAUC
5	+	AUAUUUUCGUUACUAUUUGAACCG
