# Supplementary material for: Analyses of RNA-Seq and sRNA-Seq data reveal a complex network of anti-viral defense in TCV-infected Arabidopsis thaliana
Source: Sci Rep. 2016 Oct 26;6:36007. doi: 10.1038/srep36007 (PMC5080594; doi:10.1038/srep36007)
Supplement: Supplementary Information [file srep36007-s1.pdf]

Analyses of RNA-Seq and sRNA-Seq data reveal a complex network of anti-viral defense in TCV-infected *Arabidopsis thaliana*

Chao Wu, Xinyue Li, Song Guo and Sek-Man Wong

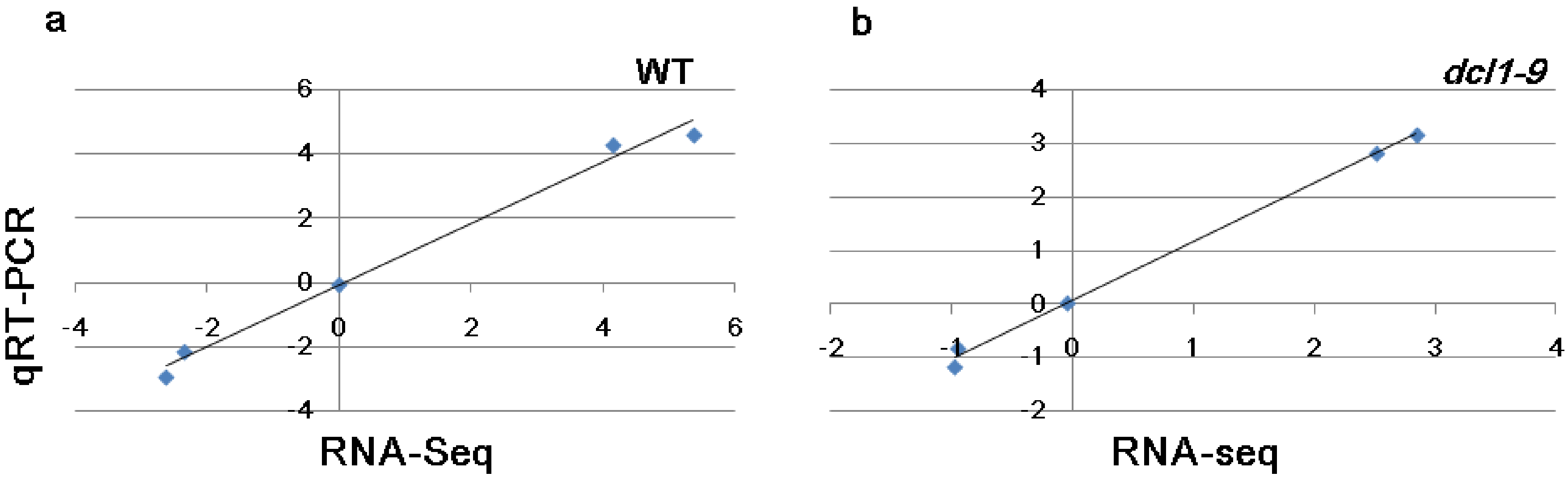

Figure S1. Validation of RNA-Seq data using qRT-PCR.
Fold changes of gene expression were detected by qRT-PCR then plotted against the data of RNA-Seq. The linear relationship between the results from qRT-PCR and RNA-Seq were indicated by the reference line.

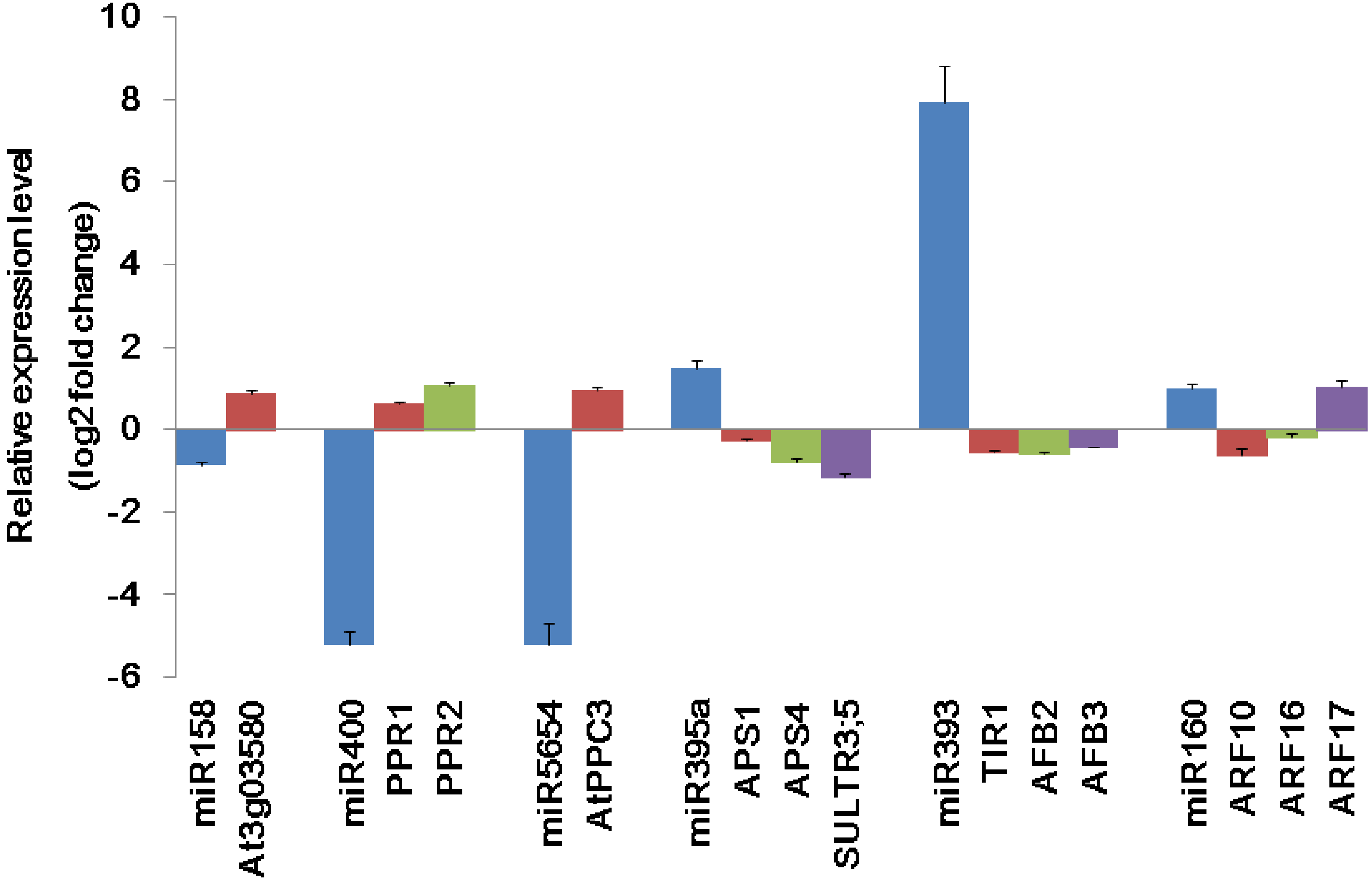

Figure S2. Expression patterns of selected miRNAs and their targets.
Relative expressions was indicated by log2 values. Most targets showed the opposite trends compared with the corresponding miRNAs.
